# Nanog interaction with the androgen receptor signaling axis induce ovarian cancer stem cell regulation: studies based on the CRISPR/Cas9 system

**DOI:** 10.1186/s13048-018-0403-2

**Published:** 2018-05-02

**Authors:** Kaijian Ling, Lupin Jiang, Shi Liang, Joseph Kwong, Leiyan Yang, Yudi Li, PingYin, Qingchun Deng, Zhiqing Liang

**Affiliations:** 1 Department of Obstetrics & Gynecology, Southwest Hospital, Third Military Medical University, Chongqing, 400038 China; 2 Bjrigham Young University, ID 272 Rigby Hall, Rexburg, 83460-4500 USA; 3 Department of Obstetrics & Gynaecology Faculty of Medicine, Prince of Wales Hospital, The Chinese University of Hong Kong, Hong Kong, China

**Keywords:** Nanog, Androgen receptor signaling axis, Ovarian cancer, Stemness properties

## Abstract

**Background:**

Ovarian cancer stem cells (OCSCs) contribute to the poor prognosis of ovarian cancer. Involvement of the androgen receptor (AR) in the malignant behaviors of other tumors has been reported. However, whether AR associates with Nanog (a stem cell marker) and participates in OCSC functions remain unclear. In this study, we investigated the interaction of Nanog with AR and examined whether this interaction induced stem-like properties in ovarian cancer cells.

**Methods:**

AR and Nanog expression in ovarian tumors was evaluated. Using the CRISPR/Cas9 system, we constructed a Nanog green fluorescent protein (GFP) marker cell model to investigate the expression and co-localization of Nanog and AR. Then, we examined the effect of androgen on the Nanog promoter in ovarian cancer cell lines (A2780 and SKOV3). After androgen or anti-androgen treatment, cell proliferation, migration, sphere formation, colony formation and tumorigenesis were assessed in vitro and in vivo*.*

**Results:**

Both AR and Nanog expression were obviously high in ovarian tumors. Our results showed that Nanog expression was correlated with AR expression. The androgen 5α-dihydrotestosterone (DHT) activated Nanog promoter transcription. Meanwhile, Nanog GFP-positive cells treated with DHT exhibited higher levels of proliferation, migration, sphere formation and colony formation. We also observed that the tumorigenesis of Nanog GFP-positive cells was significantly higher than that of the GFP-negative cells. Xenografts of Nanog GFP-positive cells showed significant differences when treated with androgen or anti-androgen drugs in vivo.

**Conclusions:**

The interaction of Nanog with the AR signaling axis might induce or contribute to OCSC regulation. In addition, androgen might promote stemness characteristics in ovarian cancer cells by activating the Nanog promoter. This finding merits further study because it may provide a new understanding of OCSC regulation from a hormone perspective and lead to the reevaluation of stem cell therapy for ovarian cancer.

**Electronic supplementary material:**

The online version of this article (10.1186/s13048-018-0403-2) contains supplementary material, which is available to authorized users.

## Background

Ovarian cancer is a lethal tumor in women worldwide, with the highest mortality rate among gynecological malignancies, and its 5-year survival rate is 30–40% depending on tumor stage [1, 2]. Although many improvements in surgical techniques and adjuvant therapies have been made, the survival rate of ovarian cancer patients has shown little improvement. The poor prognosis of this malignancy is largely due to late detection, chemoresistance, and a lack of targeted therapies for advanced and recurrent cases [2]. Evidence from epidemiological and scientific studies has shown that hormones play important roles in ovarian tumorigenesis and progression [3]. Androgen receptor (AR) is located on the X chromosome and is expressed in a diverse range of normal and cancer tissues. AR binds to its native ligand, 5α-dihydrotestosterone (DHT), with strong affinity in the nucleus. AR dimers bind to androgen response elements (AREs) in the promoter region of target genes [4]. AR has biological actions in both health and disease and participates in both normal physiological processes and pathological conditions. Several studies have shown that AR is involved in the progression of numerous malignancies, including prostate, bladder, liver, kidney and lung cancers [5, 6]. In recent years, the relationship between ovarian cancer and the AR signaling axis has become a popular topic of research because polycystic ovary syndrome and obesity are associated with a high risk of ovarian cance [7, 8]. Meanwhile, emerging evidence has indicated that AR is frequently expressed in various ovarian cancer subtypes, especially epithelial ovarian cancer, and its high expression is associated with a poor prognosis [9–13]. The AR signaling axis promotes the proliferation, migration and invasion of ovarian cancer cells in vitro and in vivo and interacts with many key signaling components, including the IL-6/IL-8 and EGFR pathways [10, 14]. Nevertheless, the mechanisms by which androgen and AR influence cancer cell growth are complex. Additionally, AR may stimulate cancer development and progression possibly by expanding the population of cancer stem cells (CSCs). CSCs are specific population of cancer cells that are responsible for tumor initiation, drug resistance and metastasis. However, the impact of AR has not been widely pursued in ovarian cancer, and to date, the precise roles of AR and CSCs in ovarian cancer are not fully understood.

There is good evidence to support the view that most human tumors harbor CSCs and that these CSCs possess the biological characteristics of normal stem cells. CSCs also possess high self-renewal, extensive proliferative and strong tumorigenic capacities, and they are the initiating cells in tumor progression, recurrence and chemoresistance [15, 16]. CSCs are able to persist in tumors and survive under nutrient starvation conditions [17]. Over the last few decades, a number of studies have identified CSCs in human ovarian cancer cells. The development and progression of ovarian cancer is fueled and sustained by these undifferentiated CSCs. Indeed, ovarian CSCs may be responsible for tumor growth, peritoneal metastasis, chemoresistance and relapse. Because CSCs can potentially arise from oncogenic reprogramming and the dynamic nature of cancer cells, identification of stem cell-related and self-renewal molecules is expected to lead to novel therapeutic targets for cancer [18].

In terms of CSC markers, Nanog has been identified as a molecule that maintains CSC pluripotency and self-renewal capability [19]. Nanog, Oct4 and Sox2 are considered pluripotent genes and stem cell markers. Nanog is a nuclear transcription factor that plays a crucial role in pluripotent cells by maintaining their embryonic stem-like properties and in cancer cells by promoting carcinogenesis and reprogramming regulation [20]. Many studies have indicated that Nanog is expressed in a variety of cancers, and its expression is correlated with poor survival [21]. Therefore, Nanog can promotes CSC properties and characteristics, and such potentially pluripotent “stem-like” cells may have an impact on tumorigenesis and progression [22]. Furthermore, Nanog is highly expressed in ovarian cancer tissues. Nanog overexpression in high-grade serous ovarian cancer is significantly associated with increased chemoresistance and poor survival. Although Nanog might function as a stem cell-associated gene involved in ovarian cancer tumorigenesis and prognosis [23, 24], the regulation of Nanog in ovarian cancer is not well understood.

Several signaling pathways that have been identified in CSCs appear to be important for maintenance of the CSC phenotype, and the participation of Nanog is a key factor [25, 26]. Meanwhile, AR has been implicated as a “molecular switch” that functions by coordinating and regulating the expression of the stem cell network. AREs contain the Nanog promoter. Thus, AR might regulate the Nanog pathway to promote the stem-like differentiation, proliferation and migration of some cancer cell types [27, 28]. In addition, our previous study confirmed that Nanog is associated with androgen/AR and plays an important role in the regulation of stemness in liver cancer cells [29]. However, these mechanisms remain unclear in ovarian cancer.

Therefore, in this study, we conducted an investigation to determine whether the AR signaling axis regulates Nanog and promotes stem-like differentiation and proliferation in ovarian cancer cells. The Clustered Regularly Interspaced Short Palindromic Repeats/protein 9 (CRISPR/Cas9) system is a simple and efficient genome-editing tool that can be applied to various cell types. We used the CRISPR/Cas9 system to target Nanog and inserted an endogenous green fluorescent protein (GFP) marker. With the CRISPR/Cas9 system, the GFP marker can directly and accurately reflect Nanog expression. Based on CRISPR/Cas9 technology, we constructed a stable and reliable cell marker model that avoids the genomic instability induced by integration of indirect genetic markers via virus vectors and the disadvantages of markers that decay over time. Hence, this approach is an efficient and stable method to examine how the androgen signaling axis regulates Nanog in ovarian cancer (Research model, Additional file 1: Figure S1). We hope to explore new treatment strategies or drug targets for ovarian cancer treatment using this technology.

## Methods

### Tissue samples and cell culture conditions

Ovarian cancer samples were collected from patients with primary ovarian cancer at the Department of Obstetrics and Gynecology. According to the protocols, all patients signed informed consent forms. This study was approved by the Institutional Review Board of Southwest Hospital, Third Military Medical University. The samples were first diagnosed by a pathologist. In total, 14 fresh samples were used for western blotting experiments: 7 were from epithelial serious ovarian cancer (stage III), and 7 were from normal ovaries. In addition, 2 epithelial serious ovarian tumors and 2 normal ovaries were used for immunohistochemistry experiments.

The human ovarian cancer SKOV3 and A2780 cell lines were purchased from the Shanghai Cell Collection. Cells were cultured in Gibco’s modified 1640 medium containing 10% fetal bovine serum (Gibco, NE), with 100 U/ml of penicillin sodium and 100 mg/ml of streptomycin sulfate (HyClone, AUS), at 37 °C in a 5% CO_2_ humidified atmosphere.

### Endogenous Nanog labeling of ovarian cells with the CRISPR/Cas9 system

The procedures for Nanog labeling via the CRISPR/Cas9 system were as follows (Flow chart, Additional file 1: Figure S2): The CRISPR/Cas9 plasmids, including PX330, were obtained from Addgene (USA), and Nanog-gRNA was designed first. Next, PX330 was digested with BbSI (New England Biolabs, UK), and Nanog-gRNA was inserted into PX330 with T4 ligase (Promega, USA). Afterwards, the plasmids were used to transform *Escherichia coli* for expansion and then extracted for Sanger sequencing. The homologous arms of the Nanog termination codon were amplified from human genomic DNA by KOD FX (TOYOBO, JPN), joined with 2A-GFP using the Gibson clone and cloned into a pMD19-T simple vector (Takara, JPN). The constructed vectors were amplified, and 2A oligodeoxynucleotide (2A-up/2A-down)/GFP sequences were synthesized and annealed before use. Then, all of these fragments were added to Gibson clone buffer (New England Biolabs, UK) to connect into loops and were used to transform competent *Escherichia coli*. Endonuclease enzyme digestion and T7E1 assays were performed as described previously. Then, the obtained products were confirmed by Sanger sequencing and PCR (Additional file 1: Figure S3, Additional file 1: Figure S4, Additional file 1: Figure S5). Therefore, we used the CRISPR/Cas9 system to label Nanog with GFP. The Nanog-2A-GFP PCR primers are listed in the Additional file 1: Table S1.

## Cell transfection

Cells were cultured for transfection in 24-well plates. When the cells reached approximately 70–80% confluence, they were transfected with 0.25 μg of PX330-Nanog-gRNA plasmid and/or 0.15 μg of the Nanog-2A-GFP homogeneous arm vector with Effectene transfection reagent (Qiagen, GER). After 72 h, GFP fluorescence was examined, and cells were sorted.

### Fluorescence-activated cell sorting

All transfected cells stably expressing GFP were sorted with a FACSAria II cell sorter (BD Biosciences, USA). Individual SKOV3 or A2780 cells with GFP expression were seeded into 96-well plates for expansion, and then labeled GFP (+) cells were verified by Sanger sequencing. Single clones of SKOV3 + 5 or A2780 + 20 GFP (+) cells and GFP (−) cells were cultured and passaged for further studies.

### RNA extraction and real-time qPCR analysis

Total RNA was extracted using an Eastep Super RNA extraction kit (Promega, USA), and then, 1 μg of RNA was converted to cDNA (reaction system 10 μl) with an Advantage® RT-for-PCR kit (Takara, JPN). After 2 μl of cDNA was mixed with SYBR Green (Bio-Rad Laboratories Ltd., USA), quantitative real-time qPCR (RT-qPCR) (reaction system 20 μl) was performed using a CFX96™ Real-Time system (Bio-Rad). GAPDH was used as the internal control. Then, we calculated the mRNA transcript abundance relative to that of GAPDH. All experiments were performed in triplicate. RT-qPCR primers are listed in the Additional file 1: Table S1.

### Western blotting analysis

For protein extraction, the tissues were first ground, and then protein was extracted with tissue lysis buffer (Thermo Fisher Scientific, USA). Cells were washed with ice-cold PBS and lysed with cell lysis buffer. Whole-cell lysates were collected from 1 × 10^6^ cells. Protein concentration was measured with a BCA protein assay kit (Beyotime, China). Western blotting assays were performed as described previously. Briefly, 50 μg of protein was loaded. The primary antibodies (1:1000) were as follows: anti-Nanog (Cell Signaling Technology, USA), anti-AR (N-20, Santa Cruz Biotechnology, USA), anti-Oct4 (Abcam, UK), anti-Sox2 (Abcam, UK) and anti-GAPDH (Cell Signaling Technology, USA). The secondary antibody was diluted 1:3000. All antibodies were used at the dilution recommended by the manufacturer.

### Immunofluorescence analysis

Cells were grown in 24-well plates that were preloaded with glass slides and cultured for 12 h. Then, the cells were fixed with 4% paraformaldehyde for 30 min and permeabilized with 0.3% Triton (Sigma Aldrich, USA) for 10 min. After blocking with 10% BSA (Sigma Aldrich, USA), the slides were incubated with primary antibodies, namely, anti-Nanog (1:200) and anti-AR (1:1000), for 16 h at 4 °C and then with the Alexa Fluor® 568-conjugated secondary antibody (1:1000) (Thermo Fisher Scientific, USA) for 1 h at room temperature. The slides were treated with DAPI (1:1000) for 15 min and mounted with 40% glycerin before examination. Immunofluorescence was detected with a confocal microscope (Carl Zeiss Jena. JER) using the following parameters: objective, 40×; scan mode, 1024 × 1024; and a scan time of 6.25 s.

### Hormone treatment

DHT and ASC-J9 were used as the drugs for hormone treatments. The drugs concentrations used were based on our previous study [29]: DHT 10 nM and ASC-J9 5 μM. Cell viability was tested using an MTS Cell Proliferation Assay kit (Promega, USA). Cells were seeded in 96-well plates at 1 × l0^4^ cells/well, cultured for 24 h, and then treated with 10 nM DHT (Dr. Ehrenstorfer GmbH, GER) and/or 5 μM dimethylcurcumin (ASC-J9) (MedChem Express, USA). Dimethyl sulfoxide (DMSO) served as the vehicle control. After further incubation for 24 and 48 h, MTT reagent (5 mg/l) was added to each well, and the cells were further incubated for 4 h. The number of viable cells was calculated relative to the appropriate controls. The mean ± SD values from three independent experiments are shown.

For hormone treatments, cultured cells were washed and placed into serum-free medium for 24 h. Then, 10 nM DHT or 5 μM ASC-J9 dissolved in DMSO was added into the medium. The cells were divided into three groups: DHT, DHT + ASC-J9 and DMSO and were harvested after 24 h.

### Luciferase reporter gene and lentivirus vector assay

Luciferase activity was detected with a Luciferase Reporter Assay System (Promega, USA) according to the manufacturer’s protocol. The luciferase backbone lentivirus was used for insertion of Nanog promoter regions with different transcriptional start site (TSS) lengths (− 500 bp, − 1000 bp, − 1500 bp). Three days after transfection, the cells were starved in FBS-free medium for 24 h and then treated with vehicle or DHT with or without ASC-J9 for another 24 h. To examine the effect of androgen on the Nanog gene promoter, the activities of different sections of the Nanog gene were tested by stimulating and (or) suppressing androgen signaling. We used constructed pGL3.0 firefly luciferase gene reporters and lentivirus vectors with three different regions of the Nanog gene.

### iCELLigence

Cell proliferation in vitro was investigated with iCELLigence software (ACEA Biosciences, USA). We sorted and treated the GFP (+) and GFP (−) cell groups treated with DHT or DHT + ASC-J9. An equal number of GFP (+)/GFP (−) cells (5 × 10^3^ cells) were seeded. GFP (+)/GFP (−) groups were divided into two subgroups: 10 nM DHT and 10 nM DHT plus 5 μM ASC-J9. The compounds were added to each well. Then, the cells were cultured and monitored for 25–37 h. All experiments were performed in triplicate.

### Transwell assay

For Transwell assays, 6.5-mm chambers with 8-μm pores (Corning, USA) were placed into 24-well plates and used to assess the migration of SKOV3 + 5 and A2780 + 20 GFP (+) or (−) cells. Cells were grown in Transwell chambers without Matrigel and treated with either DMSO, DHT or DHT + ASC-J9. An equal number of cells (5 × 10^4^ cells in 100 μl of serum-free modified 1640 medium) were seeded into the upper chambers of polycarbonate Transwell filters. A total of 600 μl of modified 1640 medium containing 10% FBS was added to the bottom of the chambers. After incubation for 16 h, migrated cells were fixed with methanol and stained with hematoxylin and eosin. Images of four random fields in each membrane were captured with a microscope. For analysis, the cells number in four fields was calculated at 40× magnification (images shown in Additional file 1: Figure S6).

### Sphere formation assay

For GFP (+)/(−) cell identification, SKOV3 + 5 or A2780 + 20 single GFP(+) or (−) cells were sorted via FACS. Then, cells were seeded into 96-well ultralow attachment plates (Corning). The medium comprised DMEM/F12 (Invitrogen, USA), 2% B27 (Prepro Tech, USA), 20 ng/ml EGF (Prepro Tech, USA), 20 ng/ml bFGF, and 4 μg/ml heparin. The cells were treated for 14 days before analysis.

### Colony formation assay

Colony formation assays were performed in 24-well plates. Cells were sorted and cultured in DMEM supplemented with 10% FBS for 14 days. In terms of hormone treatment, after seeding for 24 h, the medium was changed to DMEM with low FBS (2%), with or without the indicated reagents (DHT, DHT + ASC-J9 or DMSO). The medium was replaced every two days, and the cells were treated for 14 days. On the final day, the cells were fixed with 4% paraformaldehyde and stained with freshly prepared crystal violet for 20 min. Colony formation was observed under a microscope (Nikon, Japan).

### Immunohistochemistry

Immunohistochemical staining was performed using a conventional protocol. In brief, the tumor tissues were fixed in 4% paraformaldehyde immediately after removal from the mice. Then, samples were embedded in paraffin, sectioned, and immunostained. The sections were incubated with anti-AR (Abcam, UK) or anti-Nanog (Abcam, UK) primary antibodies at 4 °C for 16 h and secondary antibodies (DaKo, DEN) at 37 °C for 0.5 h. Finally, sections were counterstained with Mayer’s hematoxylin and mounted. Sections were visualized, and images were captured with an Olympus camera.

### Xenograft study

All animal experiments were performed in accordance with the “Guide for the Care and Use of Laboratory Animals” and were approved by the Animal Ethics Committee of the Third Military Medical University. All mice were maintained in pathogen-free conditions. Nanog A2780 + 20 GFP (+) or GFP (−) cells (5 × 10^4^) were suspended in PBS and mixed with Matrigel (volume ratio 1:1) (BD Biosciences, USA). Then, 200 μl of this mixture was injected subcutaneously into the dorsal surface of 5-week-old female nude mice. Five mice with 2 transplant sites each were used for the experiment. All mice were sacrificed after 30 days, and the tumor masses were measured. Moreover, to further validate the stemness of the GFP (+) cells and the effect of the hormone treatment in vivo, we constructed another animal model by injecting 5 × 10^3^ A2780 + 20 cells into the dorsal surface of 12 female nude mice. When the xenografts reached approximately 180 mm^3^, the animals were divided into two groups: androgen treatment and anti-androgen treatment groups. The (clinically used) drugs were as follows: the androgen was a testosterone undecanoate soft capsule (N.V. Organon, NL), and the anti-androgen was a bicalutamide tablet (AstraZeneca, UK). Intragastric drug administration was performed every three days, and the sizes of the xenografts were measured. The doses of the androgen and anti-androgen (according to the clinical reference dose) were 6 mg/kg and 8.4 mg/kg, respectively. After 9 treatments, the mice were sacrificed, and the tumors were imaged with an in vivo imaging system (PerkinElmer, USA).

### Statistical analyses

Each experiment was performed in triplicate. Statistical tests were performed using SPSS 17.0 and GraphPad Prism 5.0 (GraphPad Software). In addition, one-way ANOVA or a t-test was performed with normalization to obtain *P*-values. The criteria for statistical significance were *P* < 0.05 (*), *P* < 0.01 (**), and *P* < 0.001 (***). Relative analysis of AR and Nanog expression was calculated with Pearson’s correlation coefficient using the formula in SPSS 17.0.

## Results

### AR is overexpressed and correlated with Nanog expression in ovarian cancer

**Fig. 1:**
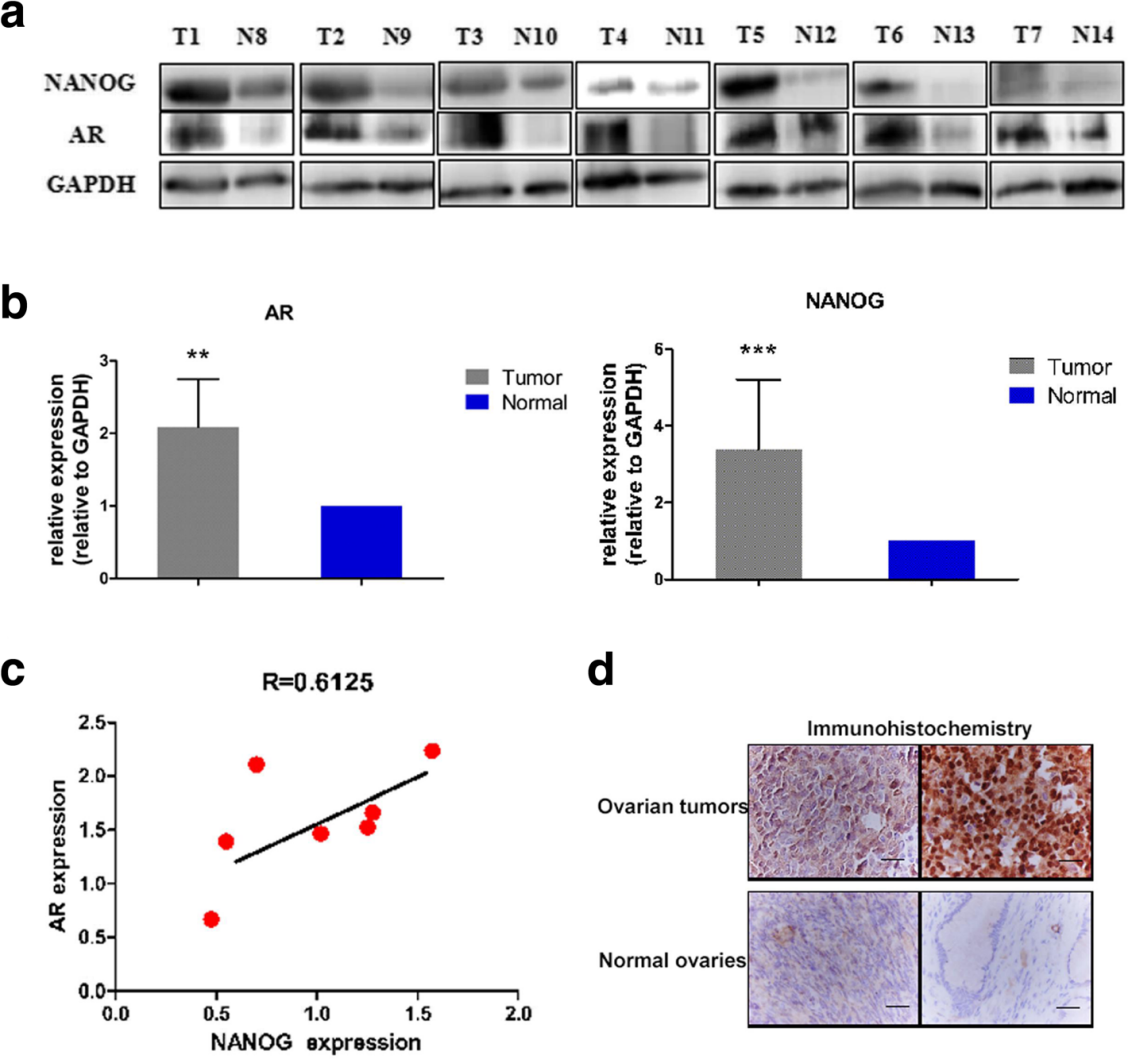
AR expression in ovarian tumors and corresponding Nanog gene expression. **a**) Western blot analysis of AR and Nanog expression in 7 ovarian tumors and 7 normal ovaries. **b**) AR and Nanog expression in ovarian tumors were higher than that in normal ovaries. Quality control software (Bio-Rad) was used to obtain the quantitative value relative to GAPDH. **c**) Correlation analysis of the AR and Nanog expression levels, indicating a correlation coefficient (R) of 0.61. **d**) Immunohistochemistry staining of AR expression in ovarian tumors and normal ovaries. In ovarian tumors, the positive (brown) staining is obvious. Bar value: 100 μM. T: ovarian tumor. N: normal ovaries. GAPDH: loading control. ** *P* < 0.01; *** *P* < 0.001

To examine AR expression in ovarian cancer, we compared the AR protein expression levels in ovarian cancer tissues (*n* = 7) with those in normal ovarian tissues (*n* = 7) with a western blot analysis. Our results showed that the AR expression levels were significantly higher in ovarian cancer than in normal ovarian tissues (Fig. 1a and b). We further confirmed AR overexpression in ovarian cancer via immunohistochemistry. Compared with the negative signals in the normal ovarian tissues, AR was strongly expressed in the cytoplasm and nucleus of ovarian tumor cells (Fig. 1d). Next, we examined the Nanog protein expression levels in the same set of samples by western blot analysis. Our results showed that the Nanog expression levels were significantly higher in the ovarian tumor tissues than in the normal ovarian tissues (Fig. 1a and b). Moreover, using Pearson’s correlation analysis, we found that the correlation coefficient (R) between AR and Nanog was 0.61 (Fig. 1c), indicating that AR and Nanog expression was correlated in ovarian tumor tissues.

### Labeling of endogenous Nanog with GFP in ovarian cancer cells with the CRISPR/Cas9 system

**Fig. 2:**
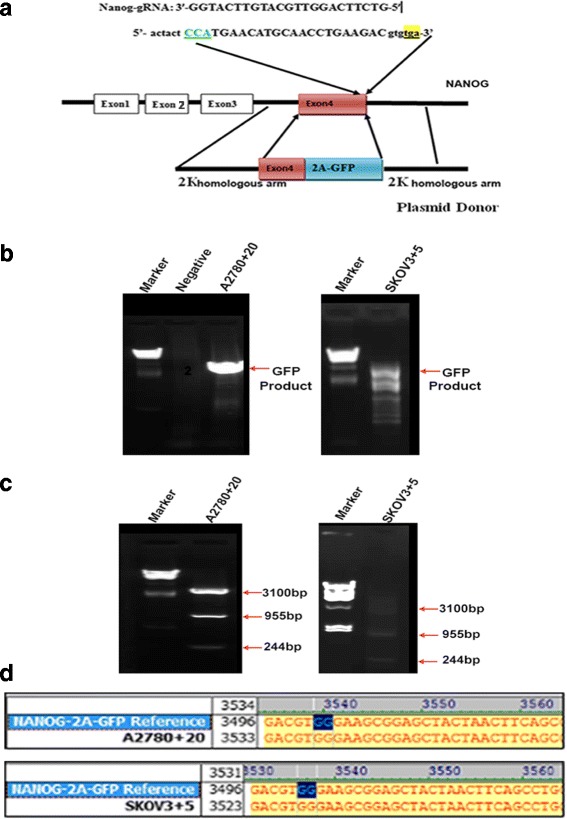
Construction and verification of an endogenous GFP Nanog marker. **a**) Map of the CRISPR/Cas9 target site on exon 4 of the Nanog gene, the gRNA sequence. **b**) The PCR GFP products of the A2780 + 20 and SKOV3 + 5 GFP (+) clonal cells (agarose gel electrophoresis). The negative control is the non-targeted A2780 cells without GFP. **c**) Enzyme digestion of PCR products from A2780 + 20 and SKOV3 + 5 monoclonal cells. **d**) The Sanger sequencing results: the bases labeled in blue represent the end of the Nanog exon 4 and the first of the 2A joint sequences in A2780 + 20 and SKOV3 + 5 monoclonal cells

To examine how endogenous Nanog expression changes under different conditions, we used the CRISPR/Cas9 system to label Nanog with GFP. Using the CRISPR/Cas9 system, we constructed PX330 Nanog-gRNA that targeted a specific site in front of the Nanog termination codon, and we established a Nanog-2A-GFP recombination homologous arm. We expected that the GFP fluorescence would represent Nanog expression in ovarian cancer cells directly and accurately. Nanog-gRNA was then inserted into the PX330 plasmid, and the Nanog gene target CRISPR/Cas9 vector, PX330-Nanog-gRNA, was generated (Fig. 2a). We co-transfected A2780 or SKOV3 ovarian cancer cell lines with the PX330-Nanog-gRNA vector and the Nanog-2A-GFP donor plasmid to generate Nanog-labeled ovarian cancer cells. Since the recombinant plasmid Nanog-2A-GFP had no promoter, the transcriptional activity of GFP appeared only when the restructured coding area was correct (Fig. 2a). Thus, the inserted endogenous Nanog GFP marker could distinguish the restructured and non-restructured cells. We obtained several single clones with the correct CRISPR/Cas9 insertion, and these clones were subsequently verified by PCR, restriction enzyme digestion, and Sanger sequencing (Fig. 2b-d). We randomly chose two independent clones, namely, A2780 clone 20 (A2780 + 20) and SKOV3 clone 5 (SKOV3 + 5), for subsequent experiments.

### AR and Nanog co-localization in ovarian cancer cells

**Fig. 3:**
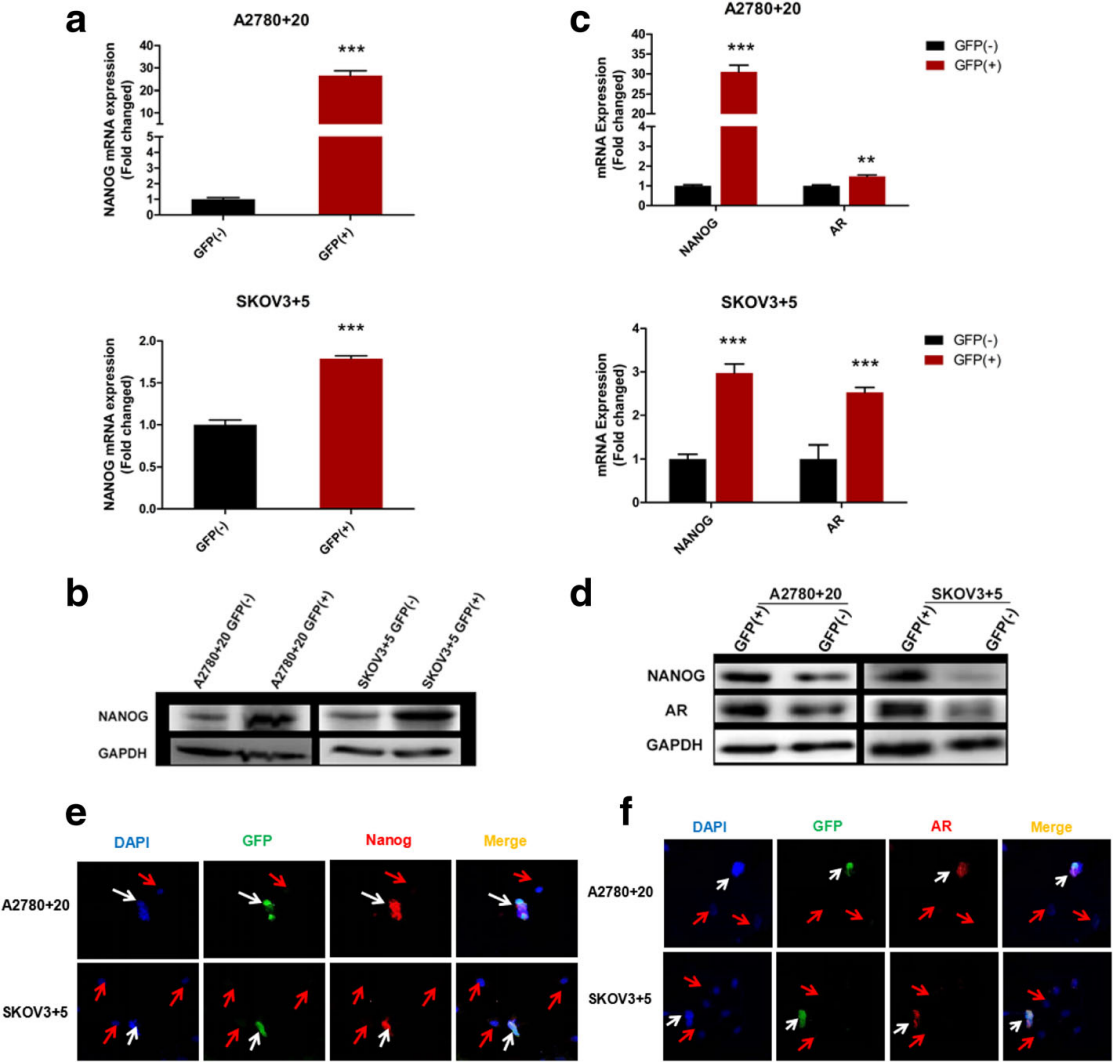
AR and Nanog co-exist in GFP Nanog (+) monoclonal cells. **a** and **b**) Nanog mRNA and protein expression in the Nanog GFP (+)/(−) cells of the A2780 and SKOV3 cell lines was examined by RT-qPCR and western blot, respectively. **c** and **d**) The mRNA and protein expression of both Nanog and AR in the Nanog GFP (+)/(−) cells of the A2780 and SKOV3 cell lines was also examined. **e** and **f**) Localization of Nanog and AR expression in Nanog GFP (+)/(−) cells determined by immunofluorescence staining; **P < 0.01; ***P < 0.001. GAPDH: loading control. Bar value: 100 μM. White arrow: double-positive cell. Red arrow: double-negative cell

GFP (+) and (−) cells from the two clones were sorted by FACS. We then determined the Nanog expression levels in the GFP (+) and (−) cells of the two clones. As predicted, Nanog expression was significantly higher in the GFP (+) cells than in the GFP (−) cells at both the mRNA and protein levels (Fig. 3a and b). To investigate possible relationships between AR and Nanog in ovarian cancer, AR expression in the GFP (+) and (−) cells of the two clones was examined. Our results showed that AR expression was significantly higher in the GFP (+) cells than in the GFP (−) cells at both the mRNA and protein levels (Fig. 3c and d). These results suggested that AR expression followed a trend consistent with Nanog expression in ovarian cancer cells. Moreover, we investigated whether the AR protein co-localized with the Nanog protein in ovarian cancer cells. AR and Nanog expression signals in the GFP (+) ovarian cancer cells were detected via confocal laser scanning microscopy. The results showed that the GFP signals were co-localized with the AR expression signals in the GFP (+) cells (Fig. 3e), indicating that GFP fluorescence accurately represented Nanog expression in the labeled cells. Furthermore, we found that the GFP signals were co-localized with the AR expression signals in the GFP (+) ovarian cancer cells (Fig. 3f). Therefore, our results indicated that AR and Nanog are co-localized in ovarian cancer cells. Moreover, a possible reciprocal action is predicted to exist.

### Androgen induces Nanog promoter activity in ovarian cancer cells through AR

**Fig. 4:**
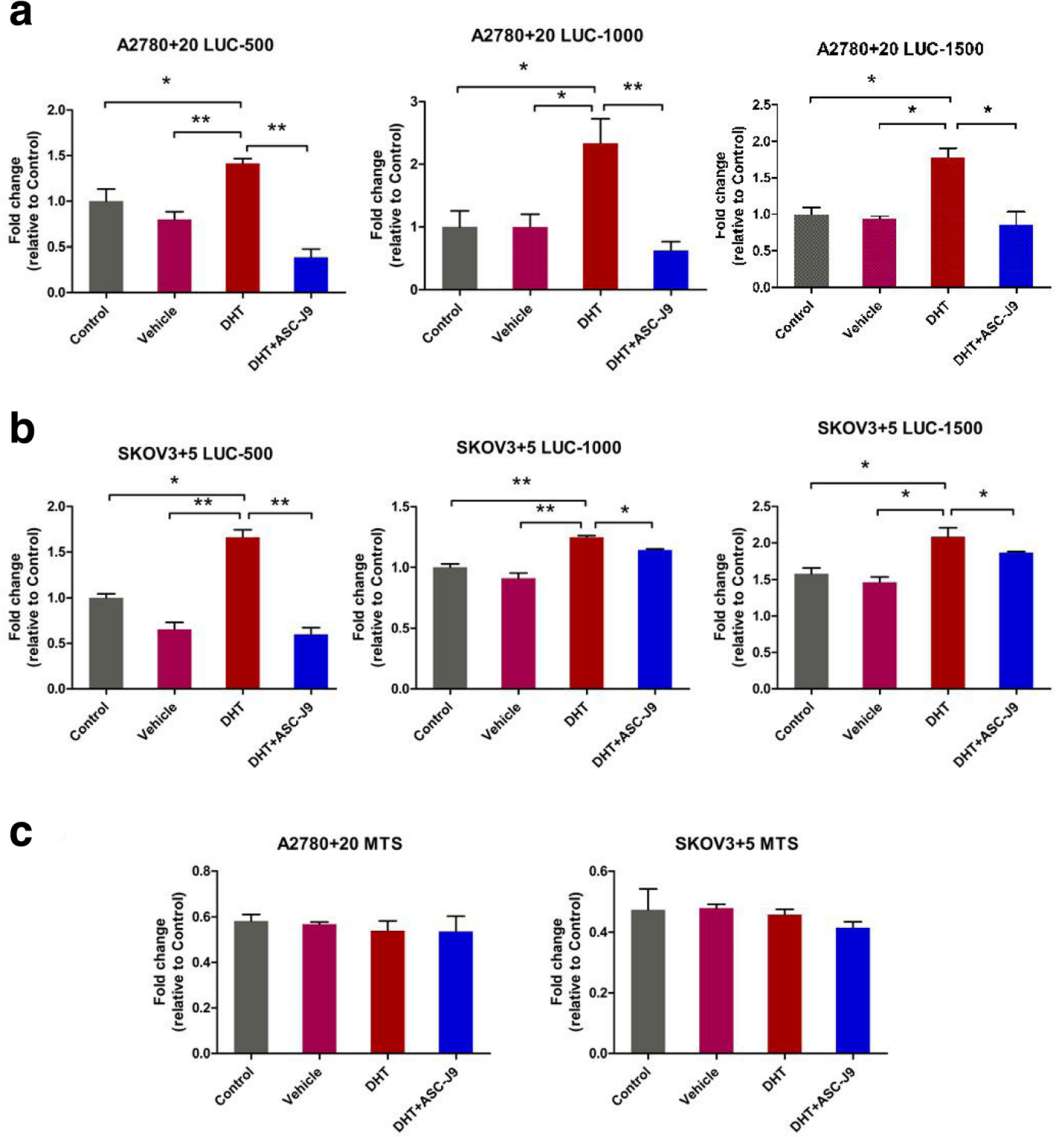
The AR signaling axis increases Nanog transcriptional activity. **a** and **b**) Promoter activities of different regions of the Nanog promoter, namely, TSS + 1~ − 500 bp, − 500~ − 1000 bp, and − 1000~ − 1500 bp, under androgen treatment in A2780 + 20 and SKOV3 + 5 cells determined using firefly luciferase reporter assays. **c**) Cell death and viability were tested with MTS assays. No differences were evident among the groups. Control: no treatment. Vehicle: DMSO; DHT: 10 nM; ASC-J9: 5 μM. *P < 0.05; **P < 0.01

Androgen up-regulates Nanog expression, and AR binds directly to AREs in the promoter region of Nanog in liver cancer cells [29]. To investigate whether androgen activates Nanog transcription in ovarian cancer cells, we performed the following promoter activity luciferase assay. We cloned three different regions of the Nanog promoter (TSS + 1 to − 500, TSS -500 to − 1000, and TSS -1000 to − 1500) into lentivirus vectors (named Lvpnanog-500, Lvpanog-1000, and Lvpnanog-1500, respectively). Each of the cloned regions of the Nanog promoter contained AREs. Using a pGL3.0 firefly luciferase gene reporter assay, we first used the lentivirus vectors containing the pGL3.0 firefly luciferase gene to transduce ovarian cancer cells (A2780 + 20 and SKOV3 + 5). To examine the effects of androgen on Nanog promoter activity in ovarian cancer cells, we treated cells with DHT or DMSO (as the vehicle control). Compared with the vehicle control, the DHT treatment significantly increased Nanog promoter activity in ovarian cancer cells (Fig. 4a and b). To confirm whether androgen-induced Nanog promoter activity was conducted through AR, we treated cells with dimethylcurcumin (ASC-J9), a selective enhancer of AR degradation. Our results showed that treatment with ASC-J9 diminished the effect of DHT on induction of Nanog promoter activity in ovarian cancer cells (Fig. 4a and b). Since treatment with DHT or ASC-J9 did not induce ovarian cancer cell death (Fig. 4c), our results indicate that androgen induced Nanog gene promoter activity in ovarian cancer cells through the AR signaling axis.

### AR signaling axis promotes ovarian cancer cell proliferation in vitro via the Nanog pathway

**Fig. 5:**
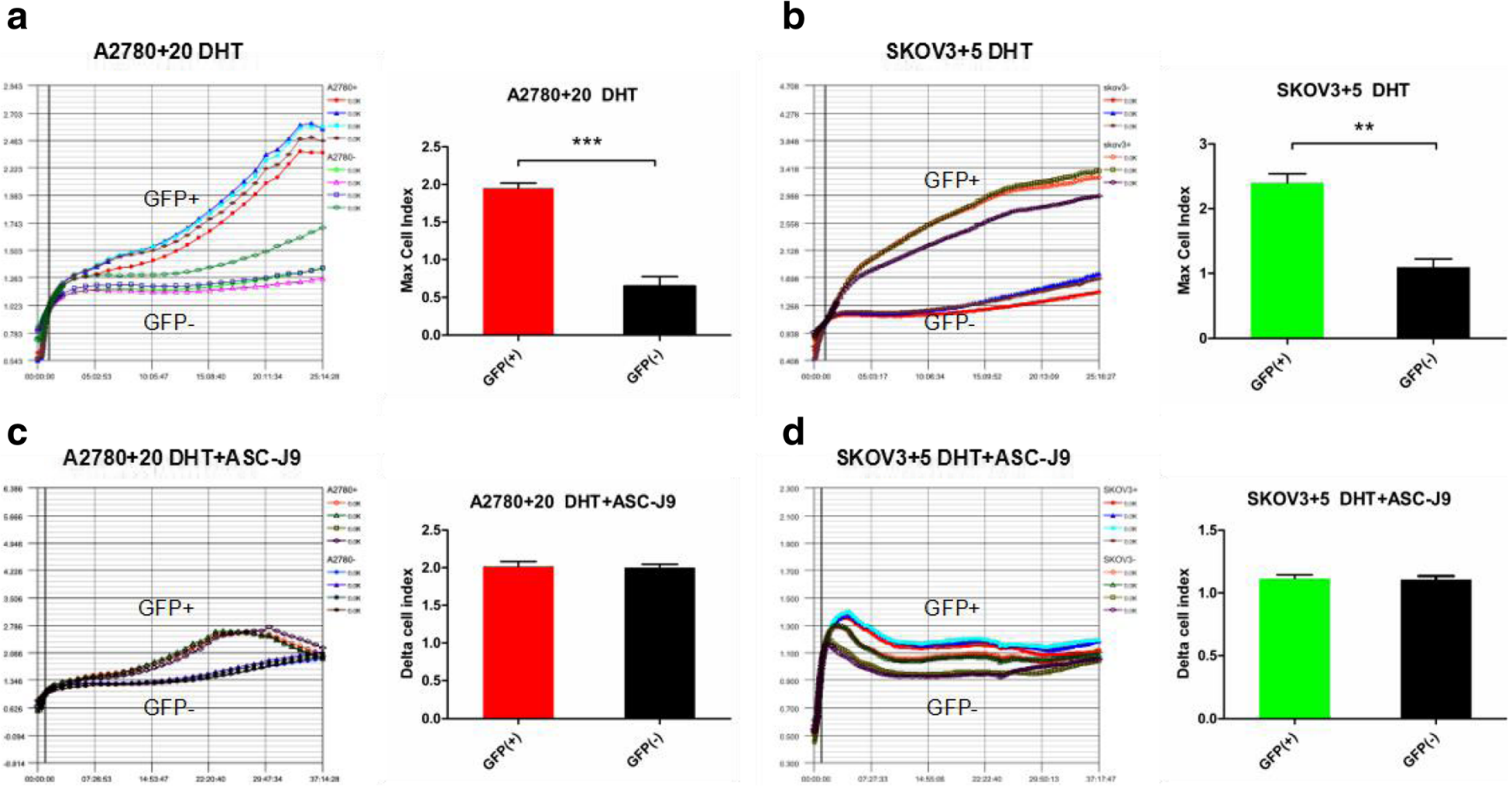
DHT-induced Nanog GFP (+) cell proliferation according to iCELLigence. **a** and **b**) The indices of the A2780 + 20 and SKOV3 + 5 GFP (+) cells treated with DHT increased more quickly than those of the GFP (−) cells within 24 h. One hour was used for normalization. The maximum cell index of the GFP (+) cells was significantly different from that of the GFP (−) cells. **c** and **d**) There were no obvious differences in the growth of the A2780 + 20 and SKOV3 + 5 GFP (+)/GFP (−) cells during treatment with DHT plus ASC-J9. The growth curves terminated within 37 h. The cell indices showed no differences. **P < 0.01; ***P < 0.001

As mentioned above, we confirmed that the dose of DHT/DMSO/ASC-J9 did not decrease cell viability. Therefore, cell growth comparisons of the DMSO-treated GFP (+) or GFP (−) cells were unnecessary. To examine whether the AR signaling axis triggered the proliferation of Nanog GFP (+) and GFP (−) cells, cell growth indices were determined using iCELLigence software. As expected, both A2780 + 20 and SKOV3 + 5 GFP (+) cells treated with DHT quickly exhibited increased cell growth compared with GFP (−) cell growth within 24 h (*P* < 0.01; Fig. 5a and b). For the DHT + ASC-J9 group, the addition of ASC-J9 attenuated the DHT effect and reduced GFP (+) cell proliferation within 36 h (Fig. 5c and d). Our results demonstrate that DHT increased cell proliferation by regulating Nanog. The results also suggested that anti-androgen (ASC-J9) might block the effect of the Nanog pathway.

### AR signaling axis increases migratory ability through Nanog activation

**Fig. 6:**
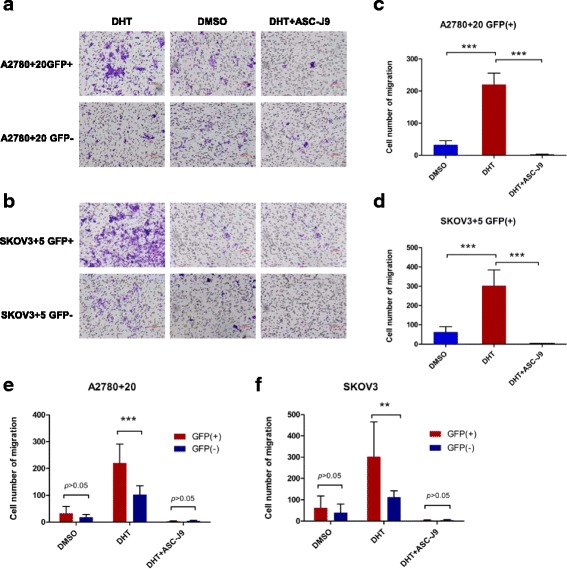
Migratory tendency of GFP (+)/GFP (−) cells when treated with different hormone drugs. **a** and **b**) The number of migratory cells increased in the DHT groups of the A2780 + 20 and SKOV3 + 5 GFP (+)/GFP (−) cell lines. **c** and **d**) Notably, when treated with DHT, the number of GFP (+) migratory cells increased markedly compared with DMSO or DHT + ASC-J9; **e** and **f**) The number of migratory in A2780 + 20 and SKOV3 + 5 Nanog GFP (+) cells were also higher than that of the Nanog GFP (−) cells. For analysis, the cells number in four fields was calculated at 40× magnification. Bar: 100 μM. DHT: 10 nM, and ASC-J9: 5 μM. **P < 0.01; ***P < 0.001

To determine whether the AR signaling axis promotes cell migration through Nanog, we examined the effect of DHT or ASC-J9 on Nanog GFP (+) and GFP (−) cells. A2780 + 20 and SKOV3 + 5 cell lines showed difference between Nanog GFP (+) and GFP (−) cells (Fig. 6a and b). Our results indicated that DHT-treated Nanog GFP (+) migrated more than cells treated with DMSO or DHT + ASC-J9 (Fig. 6b and d). Meanwhile, the migratory ability in the Nanog GFP (+) group was higher than that in the Nanog GFP (−) group (Fig. 6e and f). Taken together, these data demonstrate that the DHT-induced AR signaling axis stimulates migration through Nanog in vitro.

### Protein expression of other stem cell genes exhibits the same trend as Nanog

**Fig. 7:**
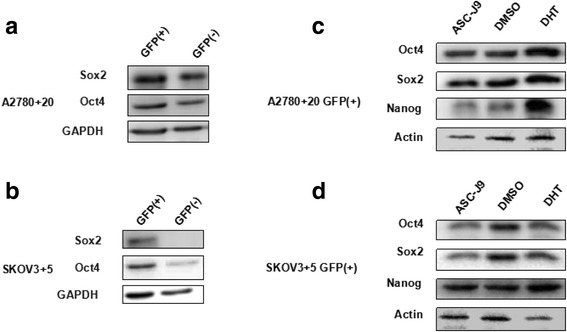
Protein expression of other stem cell genes exhibits the same tendency as Nanog. **a** and **b**) Oct4 and Sox2 expression increased in the GFP (+) cells compared with the GFP (−) cells according to western blot analysis. **c** and **d**) Oct4 and Sox2 expression in the GFP (+) cells clearly increased under DHT treatment compared with ASC-J9 treatment based on western blot analysis, which was also consistent with the Nanog expression trend

We hypothesized that Nanog protein expression was consistent with that of other stem cell genes in GFP (+) cells. Hence, we also examined the protein expression of other stem cell markers, namely, Oct4 and Sox2. Based on western blot analysis, both Oct4 and Sox2 expression in GFP-positive cells exhibited the same trend as Nanog expression (Fig. 7a and b). Moreover, we found that Oct4 and Sox2 expression increased under DHT treatment, while there were no changes under DHT + ASC-J9 treatment (Fig. 7c and d). From these results, we suspected that the AR signaling axis might promote and maintain the cell stemness phenotype.

### Androgen induces Nanog-mediated stemness properties and tumorgenicity in ovarian cancer cells

**Fig. 8:**
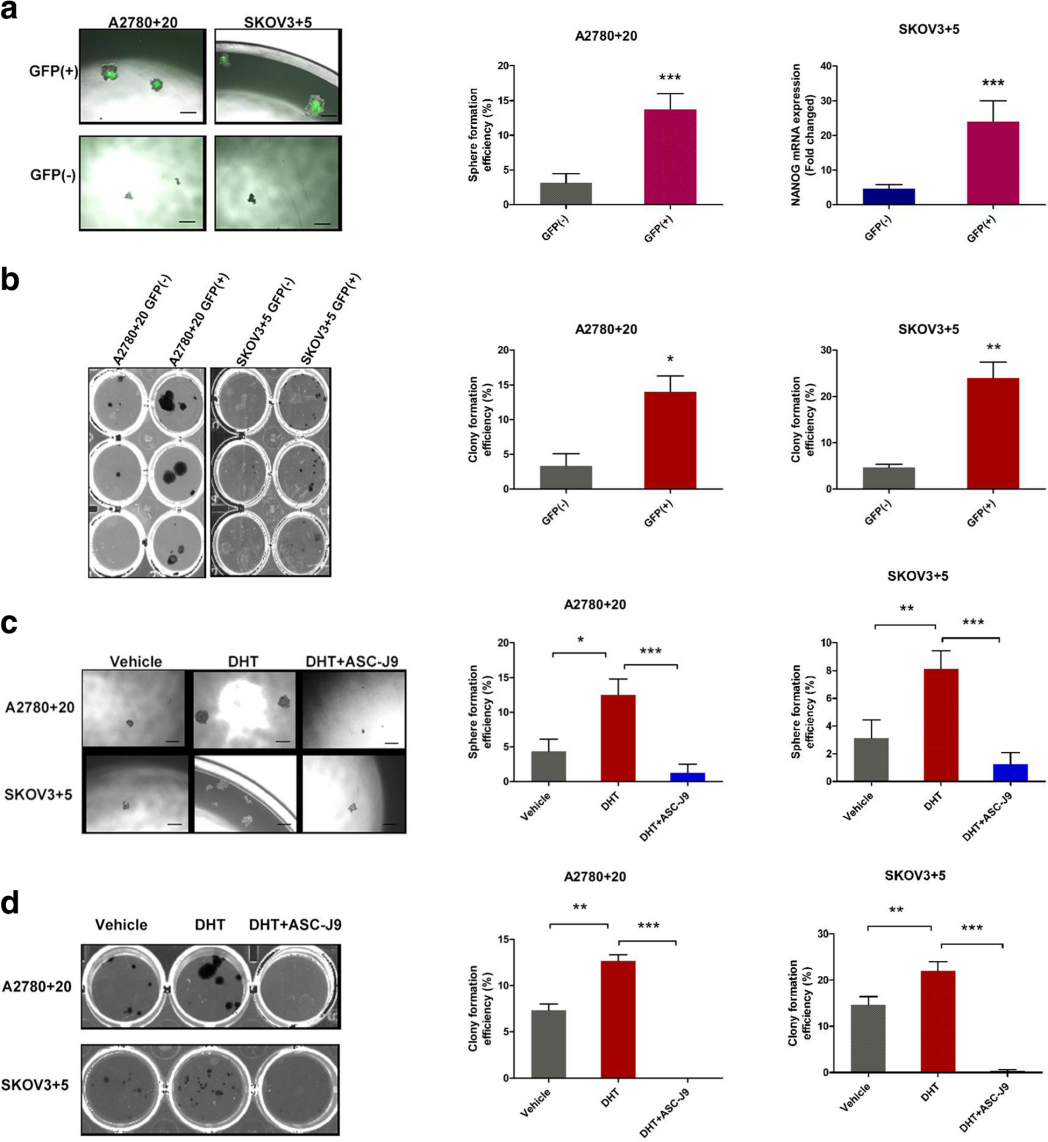
AR signaling axis enhances the stemness characteristics of ovarian cancer cells. **a**) Sphere formation assays of the monoclonal GFP (+)/GFP (−) cells of the SKPV3 + 5 and A2780 + 20 cell lines. The sphere formation abilities of the GFP (+) cell lines were significantly stronger than those of the GFP (−) cell lines. Bar: 200 μM. **b**) Colony formation assays of the monoclonal GFP (+)/GFP (−) cells of the SKPV3 + 5 and A2780 + 20 cell lines. The clonal efficiency of the GFP (+) cells was higher than that of the GFP (−) cells. Bar: 200 μM. **c** and **d**) Androgen or inhibitor treatment in SKPV3 + 5 and A2780 + 20 GFP (+) cells. Sphere and colony formation were enhanced when DHT was added, while ASC-J9 decreased this effect. DMSO was used as the vehicle control. DHT: 10 nM, and ASC-J9: 5 μM; Bar: 100 μM. *P < 0.05, **P < 0.01, and ***P < 0.001

To determine whether Nanog expression affected the stemness properties of ovarian cancer cells, we performed sphere formation assays using the monoclonal GFP (+)/(−) ovarian cancer cell lines (A2780 + 20 and SKOV3 + 5). Our results showed that the sphere formation abilities of the GFP (+) cell lines were significantly higher than those of the GFP (−) cell lines (Fig. 8a). Next, to examine whether Nanog expression affected ovarian cancer cell tumorigenicity, we performed colony formation assays using the monoclonal GFP (+)/(−) ovarian cancer cell lines (A2780 + 20 and SKOV3 + 5). Our results showed that the colony formation of the GFP (+) cell lines was significantly higher than that of the GFP (−) cell lines (Fig. 8b). These results suggested that the high Nanog expression levels in the GFP (+) cell lines promoted the stemness properties and tumorigenicity of ovarian cancer cells.

To investigate whether the Nanog-regulated stemness properties and tumorigenicity of the ovarian cancer cells were regulated by the AR signaling axis, we treated the A2780 + 20 and SKOV3 + 5 cell lines with DHT and performed sphere and colony formation assays. Our results showed that DHT treatment enhanced the sphere and colony formation of the ovarian cancer cell lines compared with the vehicle control (Fig. 8c and d). After treatment of the ovarian cancer cell lines with the AR inhibitor ASC-J9, the DHT-mediated sphere and colony formation of the ovarian cancer cell lines was diminished (Fig. 8c and d). Thus, these results suggest that androgen induces Nanog-mediated stemness properties and tumorgenicity in ovarian cancer cells through AR.

### Nanog interaction with AR promotes ovarian tumorigenicity in vivo

**Fig. 9:**
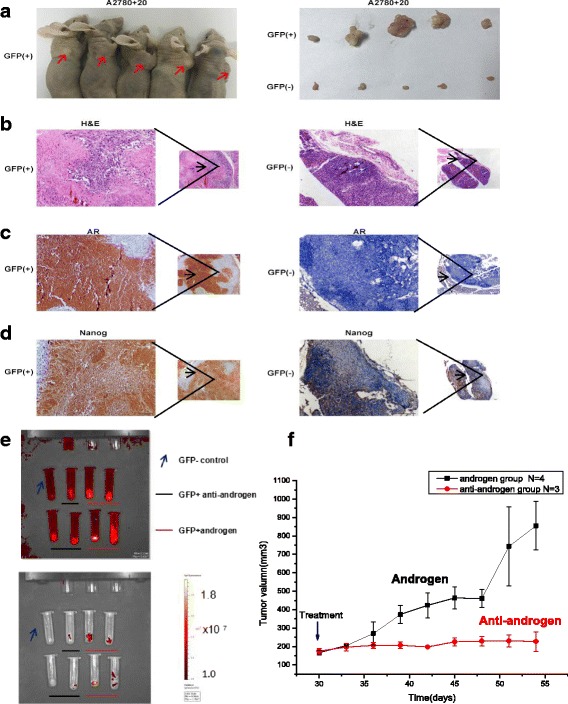
The tumorigenicity of A2780 + 20 GFP (+) cells in vivo. **a**) The xenografts produced by the A2780 + 20 GFP (+) cells were larger than those produced by the GFP (−) cells; 5 × 10^4^ cells were injected. **b**-**d**) AR expression in A2780 + 20 GFP (+) tumors was stronger than that in GFP (−) tumors. Similarly, Nanog expression was increased in A2780 + 20 GFP (+) tumors. **e** and **f**) For the in vivo hormone treatment groups, 12 mice (injected with 5 × 10^3^ A2780 + 20 GFP-positive cells) underwent xenografting, but one died before treatment. The tumor volume in the androgen treatment group increased more quickly and was greater than that in the anti-androgen group (*P* < 0.05). Fluorescence was weak after the anti-androgen treatment compared to that with androgen treatment. Photographs were captured with an in vivo imaging system

To investigate the effect of Nanog on ovarian tumorigenicity in vivo, GFP (+) and (−) monoclonal ovarian cancer cells (A2780 + 20) were inoculated subcutaneously into female nude mice to generate a xenograft model of human ovarian cancer. First, we established the animal model without hormone treatment. Our results showed that tumor xenografts formed from the GFP (+) ovarian cancer cells were significantly larger than those formed from the GFP (−) ovarian cancer cells (Fig. 9a). This result suggests that high Nanog expression levels promote ovarian cancer tumorigenicity in vivo. The AR and Nanog expression levels were higher in the GFP (+) xenografts than in the GFP (−) xenografts, determined via IHC (Fig. 9b and d). Furthermore, to explore the effect of hormone treatment in vivo, 12 mice were injected with 5 × 10^3^ A2780 + 20 GFP-positive cells to establish animal models; all mice developed xenograft tumors, but one mouse died before hormone treatment. The tumor volume in the androgen treatment group increased faster than that in the anti-androgen group. (Fig. 9f). Photographs were captured by an in vivo imaging system after the mice were sacrificed, and fluorescence in the anti-androgen treatment group was weaker than that in the androgen treatment group (Fig. 9e). Taken together, our results suggest that Nanog induces stemness properties and promotes ovarian tumorigenicity by interacting with AR in vivo*.*

## Discussion

In this study, we have demonstrated that the AR signaling axis induces Nanog-mediated stemness properties and tumorgenicity in ovarian cancer. To the best of our knowledge, few studies have addressed the relationship between AR and Nanog in ovarian cancer or the regulation of OCSCs. Several studies have reported that AR expression is higher in ovarian cancer than in normal ovaries [30]. Hence, we hypothesized that the AR signaling axis is associated with ovarian cancer. Our study began by investigating the expression levels of AR and Nanog in the same cohort of samples via IHC and western blot analysis (Fig. 1). We determined that the AR and Nanog expression levels in ovarian tumors were higher than those in the ovaries (Fig. 1a-c). AR was expressed strongly in the cytoplasm and nucleus of ovarian tumor cells (Fig. 1d). We wondered whether a direct relationship existed between AR and Nanog in ovarian cancer. Moreover, we examined whether this signaling axis was consequently involved in OCSC regulation.

Therefore, endogenous Nanog labeling in ovarian cancer cell lines was conducted to explore the association and possible mechanisms of the two genes in ovarian cancer. We generated several single clones with the correct GFP marker (A2780 + 20 and SKOV3 + 5) by using the CRISPR/Cas9 insertion system and then compared the GFP (+) and GFP (−) cells under various conditions (Fig. 2). CRISPR/Cas9 technology was used to construct a stable GFP marker for subsequent experiments that could avoid the disadvantages of other methods, such as GFP degradation. We also confirmed that AR and Nanog were co-localized in ovarian cancer cells (Fig. 3). Nanog activity was promoted by AR, which was consistent with the results of a study by Kregel et al. [27]. Meanwhile, crystallographic data have shown that AR contains an additional interface that stabilizes the AR dimer/ARE complex. In contrast, the dimerization strengths of other steroid receptors would not be sufficient to retain stable binding to selective AREs [31]. We interpreted these data as strong evidence confirming this pathway. In this study, using luciferase assays, we showed that the AR signaling axis induced Nanog promoter activity in ovarian cancer cells (Fig. 4). Ovarian cancer cells treated with different hormones were used to confirm the effect of the AR signaling axis. Moreover, to explore the possible regulation of AR and Nanog in ovarian cancer cells, the proliferation and migration abilities of single-clone Nanog GFP (+) cells with or without hormone treatments were examined (Figs. 5-6). DHT distinctly increased GFP (+) cell proliferation and migration. Under DHT + ASC-J9 treatment, no differences were evident between the Nanog GFP (+) and GFP (−) cells. Additionally, our previous study suggested that GFP (−) cells treated with DHT could undergo dedifferentiation via transformation from non-CSCs to stem-like cells [29]. For this reason, DHT not only promoted the migratory ability of the GFP (+) cells but also affected GFP (−) cells in this experiment. We also examined the Oct4 and Sox2 protein expression levels. Interestingly, Oct4 and Sox2 levels increased along with Nanog expression when cells were treated with DHT (Fig. 7). In addition, DHT clearly enhanced ovarian cancer cell sphere and colony formation compared with the vehicle control and DHT/ASC-J9 treatment (Fig. 8). Thus, our in vitro studies showed that androgen induces Nanog-mediated stemness properties and tumorgenicity in ovarian cancer cells directly through AR. Our in vivo results suggest that Nanog induces stemness properties and promotes ovarian tumorigenicity by interacting with AR (Fig. 8). This study demonstrates that the AR signaling axis interaction with Nanog induces and maintains OCSC stemness by activating the Nanog promoter. We presume that an interaction effect is present between Nanog and AR and suggest that the AR-Nanog signaling pathway participates in OCSC regulation. Even though ovarian cancer has a complex network and microenvironment, this phenomenon may mean that increased AR activity is related to a high risk of this disease. However, the evidence is not sufficient to demonstrate the mechanism of the AR-Nanog signaling axis in patients. Nevertheless, we suggest that the AR-Nanog signaling axis participates in OCSC regulation and may be a prognostic bio-marker.

We present compelling evidence to show that Nanog plays a vital role in malignant diseases and is correlated with the clonogenic growth, tumorigenicity and invasiveness of cancer cells [11, 32, 33]. Hence, Nanog appears to function as a cooperating or potentiating pro-tumorigenic molecule in the appropriate context [34]. The highlight of our study is our cell model, which was successfully built with CRISPR/Cas9 technology, and the Nanog GFP marker can be used to monitor and study authentic CSCs or pluripotency. The 2A-tdTomato sequence can reportedly be inserted by homogenous recombination to replace the stop codon of the porcine gene. Thus, fluorescence can accurately show activation of the endogenous gene through CRISPR/Cas9 [35]. These results indicate that the knock-in reporter system can be used to efficiently monitor the pluripotency status of cells. Using the CRISPR/Cas9 knock-in reporter system, we can monitor and investigate the complicated functions and regulation of genes more directly. Since the conditions of ovarian cancer cells are complex, other cell lines and networks require further elaboration.

Previous studies have not demonstrated efficient effects when anti-androgen is used to treat patients, but we should still consider the effects and design more animal studies to investigate this process [36]. In the future, we hope to identify new anti-androgen approaches to eliminate OCSCs or to inhibit cancer cell growth in patients with high AR expression.

## Conclusion

In conclusion, this study demonstrates that AR functions as an oncogene by promoting Nanog expression in ovarian cancer cells, and the interaction of Nanog with the AR signaling axis might induce or contribute to OCSC regulation. Androgen might promote stemness characteristics in ovarian cancer cells by activating the Nanog promoter. These findings may provide a new understanding of OCSC regulation from a hormone perspective and lead to the reevaluation of stem cell therapy for ovarian cancer.

## Additional file

Additional file 1: Supplementary methods and materials. (PDF 1433 kb)

## Abbreviations

AR: Androgen receptor
AREs: Androgen response elements
ASC-J9: dimethylcurcumin
CRISPR/Cas9: Clustered Regularly Interspaced Short Palindromic Repeats/protein 9
DHT: 5α-dihydrotestosterone
DMSO: Dimethyl sulfoxide
FACS: Fluorescence-activated cell sorting
GFP: Green fluorescent protein
OCSCs: Ovarian cancer stem cells
