# Correction to: Nanog interaction with the androgen receptor signaling axis induce ovarian cancer stem cell regulation: studies based on the CRISPR/Cas9 system

**DOI:** 10.1186/s13048-019-0487-3

**Published:** 2019-01-30

**Authors:** Kaijian Ling, Lupin Jiang, Shi Liang, Joseph Kwong, Leiyan Yang, Yudi Li, PingYin, Qingchun Deng, Zhiqing Liang

**Affiliations:** 1 Department of Obstetrics & Gynecology, Southwest Hospital, Third Military Medical University, Chongqing, 400038 China; 2 Bjrigham Young University, ID 272 Rigby Hall, Rexburg, 83460–4500 USA; 3 Department of Obstetrics & Gynaecology Faculty of Medicine, Prince of Wales Hospital, The Chinese University of Hong Kong, Hong Kong, China

**Correction to: J Ovarian Res (2018) 11:36**

**https://doi.org/10.1186/s13048-018-0403-2**

The original article [1] contains errors in Figs. 6 and 8. The corrected figures can be shown ahead.

**Fig. 6:**
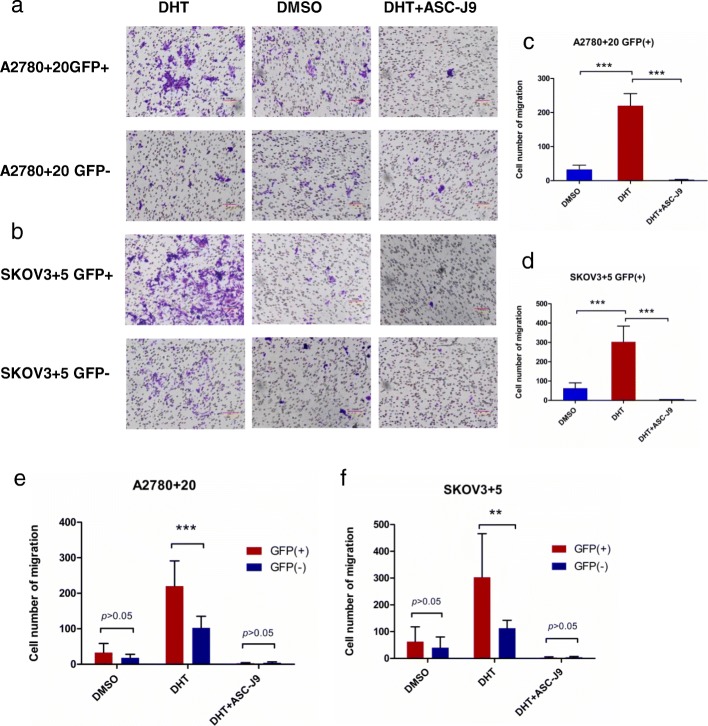
Migratory tendency of GFP (+)/GFP (−) cells when treated with different hormone drugs. **a** and **b**) The number of migratory cells increased in the DHT groups of the A2780 + 20 and SKOV3 + 5 GFP (+)/GFP (−) cell lines. **c** and **d**) Notably, when treated with DHT, the number of GFP (+) migratory cells increased markedly compared with DMSO or DHT + ASC-J9; **e** and **f**) The number of migratory in A2780 + 20 and SKOV3 + 5 Nanog GFP (+) cells were also higher than that of the Nanog GFP (−) cells. For analysis, the cells number in four fields was calculated at 40× magnification. Bar: 100 μM. DHT: 10 nM, and ASC-J9: 5 μM. **P < 0.01; ***P < 0.001

**Fig. 8:**
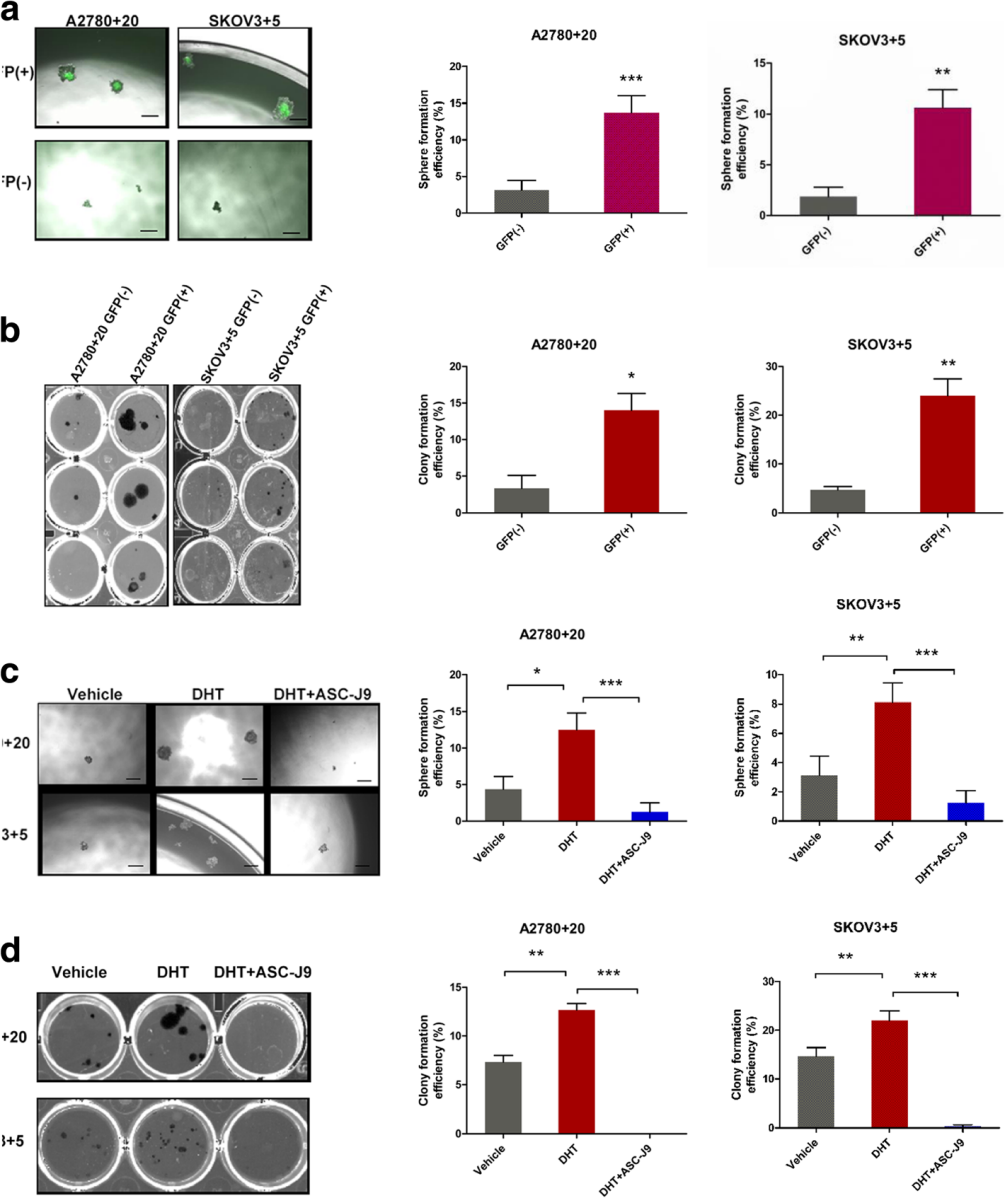
AR signaling axis enhances the stemness characteristics of ovarian cancer cells. **a**) Sphere formation assays of the monoclonal GFP (+)/GFP (−) cells of the SKPV3 + 5 and A2780 + 20 cell lines. The sphere formation abilities of the GFP (+) cell lines were significantly stronger than those of the GFP (−) cell lines. Bar: 200 μM. **b**) Colony formation assays of the monoclonal GFP (+)/GFP (−) cells of the SKPV3 + 5 and A2780 + 20 cell lines. The clonal efficiency of the GFP (+) cells was higher than that of the GFP (−) cells. Bar: 200 μM. **c** and **d**) Androgen or inhibitor treatment in SKPV3 + 5 and A2780 + 20 GFP (+) cells. Sphere and colony formation were enhanced when DHT was added, while ASC-J9 decreased this effect. DMSO was used as the vehicle control. DHT: 10 nM, and ASC-J9: 5 μM; Bar: 100 μM. *P < 0.05, **P < 0.01, and ***P < 0.001
