# Supplementary material for: Nanog interaction with the androgen receptor signaling axis induce ovarian cancer stem cell regulation: studies based on the CRISPR/Cas9 system
Source: J Ovarian Res. 2018 May 2;11:36. doi: 10.1186/s13048-018-0403-2 (PMC5930492; doi:10.1186/s13048-018-0403-2)
Supplement: Supplementary file 1 — Supplementary methods and materials. (PDF 1433 kb) [file 13048_2018_403_MOESM1_ESM.pdf]

Supplementary methods and materials

Figure S1

Research model map

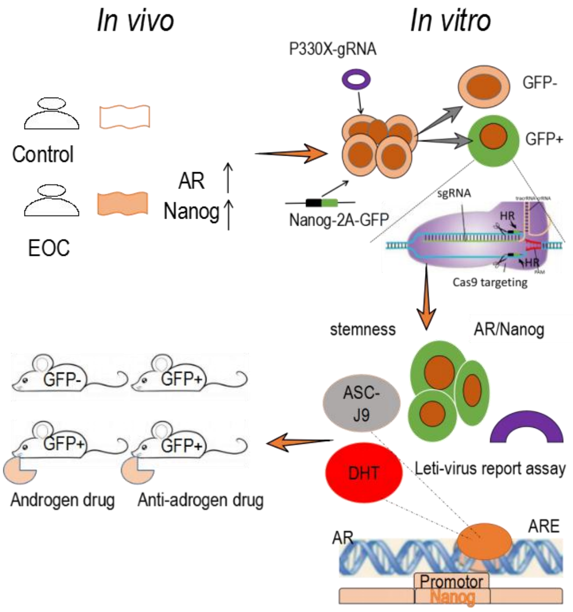

Figure S2

Flow-chart for gene manipulation protocol

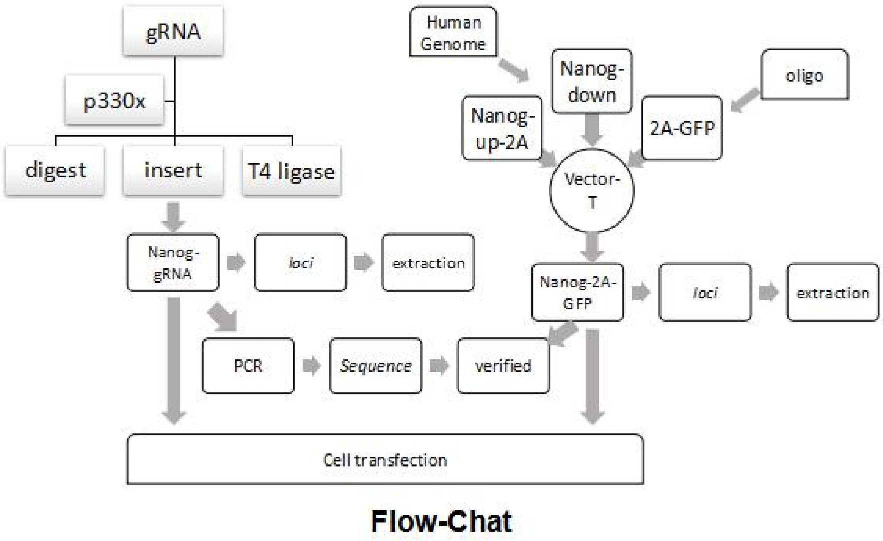

Constructed CRISPR/ Cas9-gRNA plasmid

First, we designed a single guide RNA (gRNA) targeted to the Nanog termination codon (http://crispr.mit.edu/) as follows: Nanog-gRNA-F1, CACCGTCTTCAGGTTGCATGTTCA and Nanog-gRNA-R1, AAACTGAACATGCAACCTGAAGAC. The primers were synthesized by Jingsirui. We

obtained the gRNA with the following PCR reaction parameters: 4 µl of each primer (100 nM) for Nanog-gRNA (F+R) was placed into the mixture with 4 µl of buffer + 12 µl of ddH_2O. The primer annealing procedure was 95°C for 2 min, followed by 95°C→25°C (down 0.1°C every 8 seconds for 700 cycles), with a 12°C hold thereafter. Second, the P330X plasmid with Cas9 expression packaged in the loci was transformed into *Escherichia coli*, expanded and extracted. Next, we prepared the P330X plasmid enzyme as follows: 5 µl of 10x buffer + 3 µl of PX330 (1 µg/µl) + 1 µl of BbSI endonuclease + 11 µl of ddH_2O. We then performed gRNA conjugation as follows: 3 µl of PX330 (1 µg/µl) + 1 µl of gRNA + 1 µl of T4 DNA ligase + 1 µl of T4 ligase buffer + 4 µl of ddH_2O at 16°C for 3 hours. Loci transformation was subsequently performed. The products were verified by PCR and Sanger sequencing (Supplementary Fig. 1). The PCR reaction (25 µl) comprised the following: 2.5 µl of 10× LA Taq buffer + 4 µl of dNTP + 0.25 µl of LA Taq + 2 µl of Primer Mix + 1 µl of PX330-Nanog-gRNA (988 ng/µl) + 15.75 µl of ddH_2O. The PCR procedure was as follows: 94°C for 3 min, followed by 35 cycles of 94°C for 30 s→60°C for 30 s→72°C for 30 s, with a 12°C hold thereafter.

Construction of the 2A-GFP homologous recombination arm plasmid

Genome extraction was performed with human blood, and the upstream and downstream 2Kb sequences of the Nanog gene termination codon were amplified by PCR (Table S1). Nanog-T-vector, Nanog-2A-up, Nanog-down and 2A-GFP sequences were amplified separately. The reaction was performed as follows: 2 µl of 10x buffer + 1 µl of DPNI enzyme + 15 µl of PCR products + 2 µl of ddH_2O in a 37°C water bath for 5 hours.

Nanog-2A-up, Nanog-down and 2A-GFP were ligated There was a 20-bp overlap, The Nanog-T-vector amplification had a 30-bp sequence overlap between the upstream and downstream regions. Finally, we used the Gibson cloning method for large DNA fragment ligations. The Gibson cloning method was performed in a total volume of 20 µl as follows: 0.5 µl of Nanog-2A + 1 µl of 2A-GFP + 1.5 µl of Nanog-down + 2 µl of Nanog-T-vector + 15 µl of the master mix at 50°C for 1 hour. The loci transformations of the Gibson products, PCR identification and Sanger sequencing were performed (Supplementary Figs. 3-5).

Figure S3

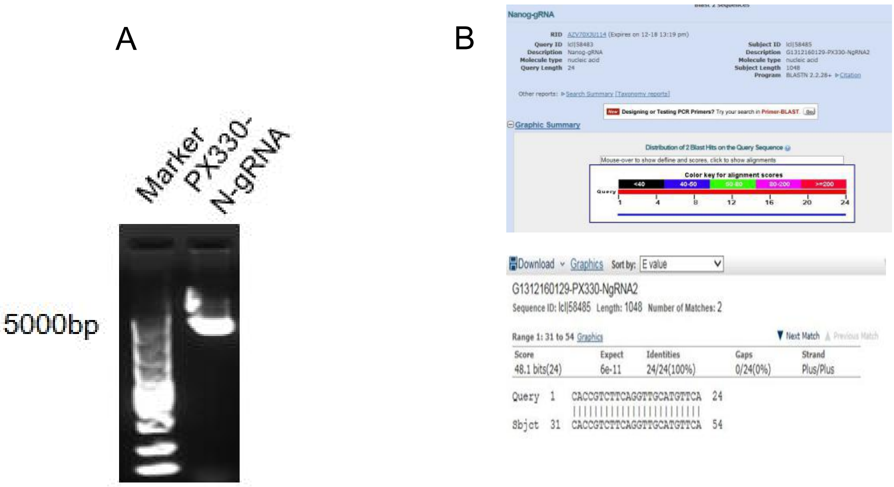

PX330-Nanog-gRNA electrophoresis and Sanger sequence documents

A. PX330-Nanog-gRNA ；
B. B.PX330-Nanog-gRNA Sanger sequencing documentary proof

Figure S4

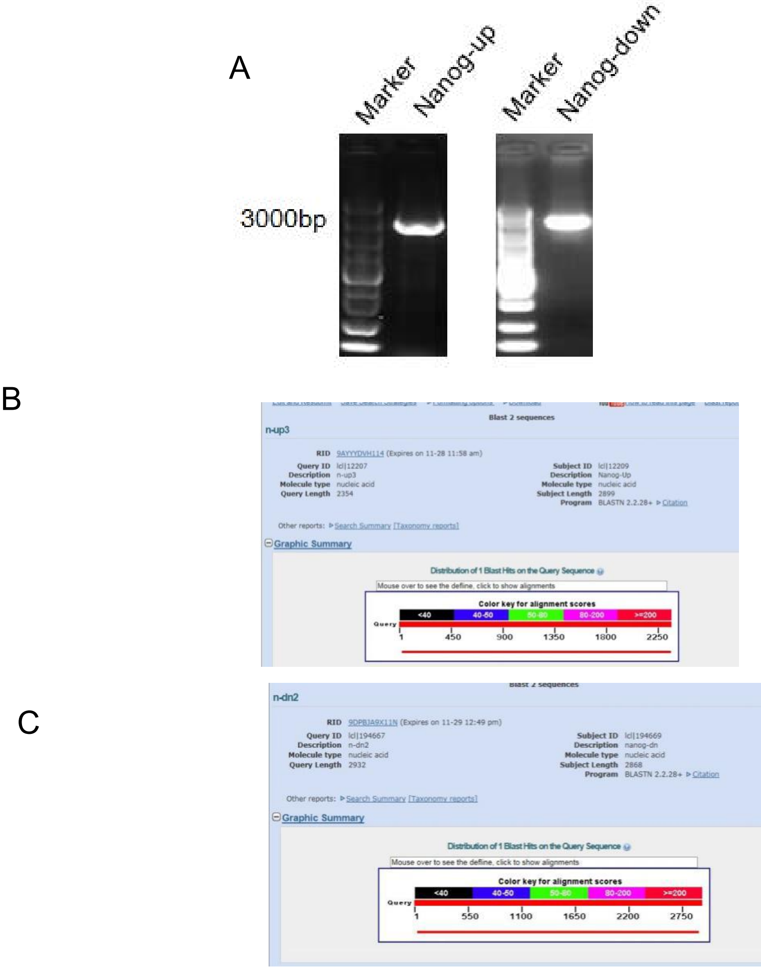

Nanog 2kb recombination arm PCR product and Sanger sequence documents

A. Nanog-up and Nanog–down 2kb PCR electrophoresis；

B. Nanog-up recombination arm 3（N-up3）Sanger sequencing documentary proof；

C. Nanog–down recombination arm 2（N-dn2）Sanger sequencing documentary proof.

Figure S5

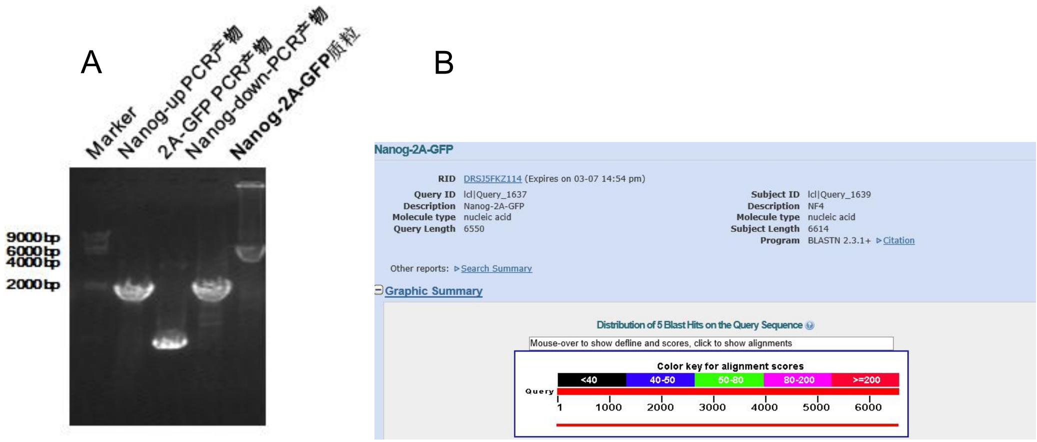

Nanog-2A-GFP recombination arm PCR product and Sanger sequence document

A. Nanog-2A-GFP PCR product electrophoresis，Nanog-up：Nanog-up recombination arm，Nanog-down： Nanog–down recombination arm， 2A-GFP： 2A-GFP PCR product.

B. Nanog-2A-GFP documentary proof.

Endonuclease enzyme digestion and T7E1 assay

Because Nanog-2A-GFP has no promoter, the transcriptional activity of the GFP can only be accessed while restructuring the Nanog coding rear area. Next, to verify Nanog-2A-GFP by PCR, we designed the upstream primer and downstream primer binding sites on the GFP-Nanog joint sequence. With extra HindIII enzyme *loci* sites on the GFP sequences, the correct target cell PCR amplification products comprised three HindIII enzyme bands after digestion with the restriction HindIII enzyme.

Primers listed below

Table S1 The PCR primers for the 2A-GFP homologous recombination arm

Name	Sequence	Sequence – (5’-3’)
Nanog-up primer	Nanog-up-F: TGAGGTGCTGGTTTTATAGAATCCCCA	Nanog-up-R: CACGTCTTCAGGTTGCATGTTCATGG
Nanog-down primer	Nanog-down-F AGATGAGTGAAACTGATATTACTCAATTTCAGTC	Nanog-down-R AACATAATACAGGGCTAGGCTGGTGAC
Verified primer	Primer-up	Primer-down

	AGAGCCTGTCCCTTTGTTATGTGACTG	TGGACGAGCTGTACAAGTAAAGATGA GTGAAA- CTGAT ATT)

Table S2 Primer for RT-qPCR

Gene primer name	Sequence (5'-3')
RT-Nanog primer	Forward: CTCTCCTCTTCCTTCCTCCAT
	Reverse: TTGCGACACTCTTCTCTGC
RT-AR primer	Forward: TAGCCCCCTACGGCTACA
	Reverse: TTCCGAAGACGACAAGATGGAC
RT-GAPDH primer	Forward: CACCCACTCCTCCACCTTTG
	Reverse: CCACCACCCTGTTGCTGTAG

Figure S6

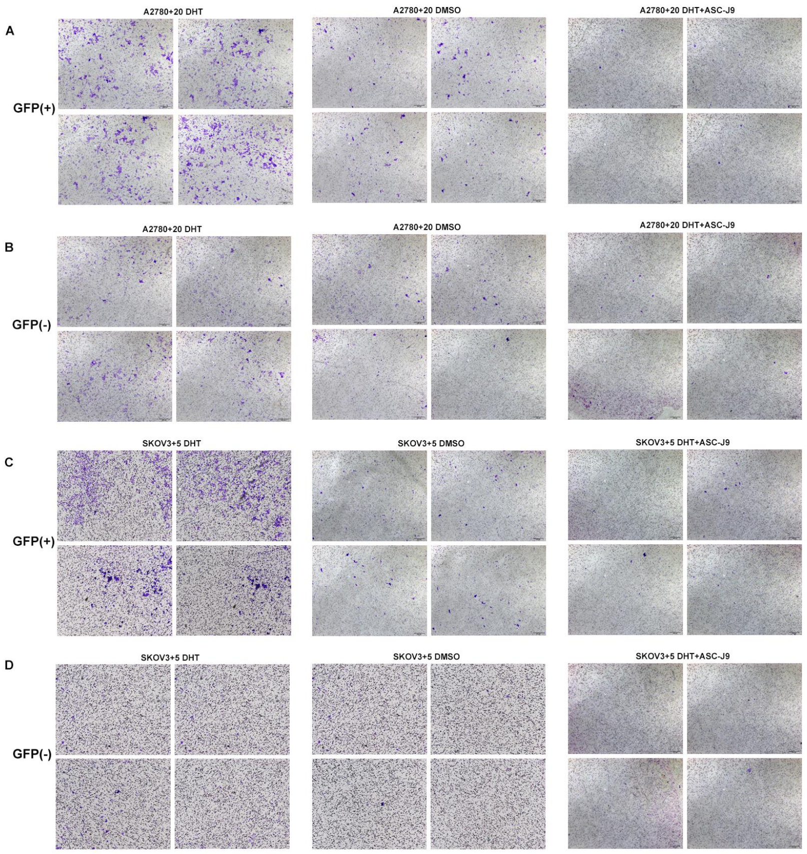

Migratory tendency of GFP (+)/GFP (-) cells with hormone treatment

The number of migratory cells increased in the DHT groups of the A2780+20 and SKOV3+5 GFP (+)cell lines（Fig A and C) when compared with GFP (-)cell lines (Fig B and D). Magnification:40X; Bar scale: 200 μM.

Nanog promoter sequence file

cgatcgtacgcctgcccatccctcccctaccccaacctccattatttgtactctcttagcatccagtgcactttttttccttact

tattacacaactttacacttacaattaacattataattgtctttttggtctatttgatctactctactaaatctttttttttttt

tttgagacaaggtctcactgtgcctcctcggctggagtgcagtggcgtgatctcggctcgctgcaacctccgcctctggag

ttcaagtgattctcctgtctcagcctccctagtagatgggattacaggcgtgcaccaccacgcccagcccagttaattttg

tatttttagtacagacagggtttcaccatgttggtcaggctggtcttgaactcctgatctcagatgatgcacctggctcggc

ctcgcaaagtgctgggattacaggtgtgagccaccgtgcccagccgttagctcattttaacacatccttagtccagcctgt

tccaaaaaatctaaagtcagatagcttcctaaacctcaactttattccaattgctttccttggcgaagaatgtagtaagtc

ggccttccagccaccagccccttccctttggtctttcactccggaggctcttaccctagacacaatgggacagggagcgg

gggatgggggaattcagctcaggcttttatgcaaagaccccccttctgcaaagaacaaagcttctggtacctgccctttgg

agagctgcgggcaagctcagcctcggtgagtcttggtggccttgacagcccccacttaacaaactgtgctgattaagag

agacaggagggcaagtttttcccttcttttaaagaaatcatcctatttcctacgagacatagactatctgcctgaagcatg

atgtactagccccactcaccggctccctgatgcccctatgcttaatcttctccggaatggtagtctgagaagaaaaaaga

ttacgcccaatttcatttccttgtttcacatcaagcaatacttttcgagtctttgcattgtgaacaaaagtcagcttgtgtgg

gagcaaagccagctgctctgggtgcagacccaggagcagagtgcagaggagaatgagtcaaagagttttgtcttcaaa

aattacataatcgggatttgctaagagtttacttttcggtatggaagactggaaaagagaaagaaatcttaggtttcttg

aatgttgggtttgggaataggaaggaaaatcgaaaactgtagactttgtccataaatgttagtgctggaaccccactct

aaaaactttgttcctttggaaaacacctcccttcccccagaaacacacacacccacacgagatgggcacggagtagtct

tgaaagacatgacaaatcaccagacctgggaagaagctaaagagccagagggaaaaagccagaagtcgactacctg

ggaggagggatagacaagaaaccaaactaaaggaaactaaggtaggtgctgaaaacaagtaccattttcaacatta

actgatgccttggcttcatgctataatgccatgttgtgtttcactataacctcagagtgaatgaaagaggaaaatggagc

tagttgaaatttctgcctaaactagccagattttgagacactaagttatctcaaatcaagaaatcaccctaatgagaattt

caataacctcaggaatttaaggtgcatgcatcccccacccccccctttttttttgagacgtagtcccgctctgttgcccag
